# Dengue Virus Infection in Sub-Saharan Africa Between 2010 and 2020: A Systematic Review and Meta-Analysis

**DOI:** 10.3389/fcimb.2021.678945

**Published:** 2021-05-25

**Authors:** Khalid Eltom, Khalid Enan, Abdel Rahim M. El Hussein, Isam M. Elkhidir

**Affiliations:** ^1^ Department of Microbiology and Parasitology, Faculty of Medicine, University of Khartoum, Khartoum, Sudan; ^2^ Department of Virology, Central Laboratory, Ministry of Higher Education and Scientific Research, Khartoum, Sudan

**Keywords:** dengue (DENV), Sub-Saharan Africa (SSA), systematic review, meta-analysis, epidemiology

## Abstract

Dengue virus (DENV) infection has garnered a global interest in the past few decades. Nevertheless, its epidemiology in certain developing and low-income regions remains poorly understood, due to the absence of comprehensive surveillance and reporting systems. This systematic review and meta-analysis aimed to determine the prevalence of DENV infection in the population of Sub-Saharan Africa using DENV infection markers, and to track any changes in its prevalence during the past ten years. It was conducted in accordance with the PRISMA guidelines, targeting the literature available at MEDLINE/PubMed, ScienceDirect, Cochrane library and Google Scholar. All articles published in English language between January 2010 and June 2020 were screened for eligibility. Random effects model was used to calculate the pooled prevalence of all infection markers. The Inconsistency Index (*I* ^2^) was used to assess the level of heterogeneity between studies. Subgroup analysis according to country and time-frame of studies was conducted to provide possible explanations to substantial heterogeneity. The critical appraisal tool for prevalence studies designed by the Joanna Briggs Institute (JBI) was used to assess the risk of bias in all included studies. A total of 84 articles, covering 21 countries, were included in this review. Quantitative meta-analysis estimated a pooled IgG prevalence of 25% (95% CI: 21-29%, *I* ^2 ^= 99%), a pooled IgM prevalence of 10% (95% CI: 9-11%, *I* ^2 ^= 98%) and a pooled DENV RNA prevalence of 14% (95% CI: 12-16%, *I* ^2 ^= 99%). Evidence for possible publication bias was also found in all three meta-analyses. Subgroup analysis according to the time of sample collection was performed to closely track the changing prevalence of DENV infection markers between 2010 and 2019. This meta-analysis estimates a high prevalence of DENV infection in Sub-Saharan Africa. More cost-efficient vector control strategies should be designed and implemented in order to adapt to the low-resource nature of this region.

## Introduction

Dengue virus (DENV) is an arthropod-transmitted, positive stranded RNA virus of the family *Flaviviridae* and the genus *Flavivirus*. There are four distinct serotypes of Dengue virus (DENV 1-4), all of which are transmitted by *Aedes* mosquitos, mainly *Aedes aegypti* and to a lesser degree by *Aedes albopictus* (World Health Organization, 2014; Guzman et al., 2016). Since its first outbreak in Jakarta, Indonesia in 1779, dengue fever outbreaks remain a serious public health concern. The World Health Organization (WHO) estimates that 100-400 million DENV infections occur annually, with an 8-fold increase in global incidence observed over the past 20 years; from 505,430 cases in 2000, to over 2.4 million in 2010, and 4.2 million in 2019. A marked increase in fatalities due to DENV infections was also noted in certain regions of the world such as the Eastern Mediterranean Region, where the reported deaths increased from 960 in 2000 to 4032 in 2015 (World Health Organization, 2014).

DENV infections can be asymptomatic or relatively mild, however, the clinical spectrum of DENV infections extends to involve more severe and possibly fatal manifestations such as shock and/or fluid accumulation (with or without respiratory distress), severe bleeding and severe organ impairment (WHO, 2009). Other complications of DENV infections such as encephalitis, myelitis and fulminant hepatitis were also reported (Ling et al., 2007; Wasay et al., 2008). The pathogenesis of severe DENV infections has been a subject of study for decades and a number of virus-related risk factors have been identified, the most important of which is the antibody-dependent enhancement (ADE) of secondary DENV infections when caused by heterologous DENV serotypes. Several host-related risk factors, such as the ethnicity and genetic makeup of patients, are also reported to affect the clinical course of the disease (PP et al., 1987; Chaturvedi et al., 2006).

Due to its international epidemiology, DENV infections should be monitored globally, and its outbreaks rapidly identified to minimize the resulting morbidity and mortality. Nevertheless, low-income regions - such as Sub-Saharan Africa - are known to lack the necessary resources and infrastructure needed for proper surveillance of this disease. This systematic review and meta-analysis aims to identify all DENV-related prevalence studies conducted in Sub-Saharan Africa in the past decade, in order to estimate the pooled prevalence of DENV infection markers (DENV-specific Immunoglobulin G (IgG), Immunoglobulin M (IgM), and DENV RNA) in this region, and to track any changes in these prevalence estimates during the last ten years in an effort to attain a comprehensive understanding of DENV epidemiology in Sub-Saharan Africa.

## Methods

This systematic review and Meta-analysis was conducted in accordance with the Preferred Reporting Items for Systematic reviews and Meta-Analyses (PRISMA) guidelines (Liberati et al., 2009) (**Supplementary Table 1**). The review protocol was registered on Open Science Framework (OSF Registries) under the following doi: (10.17605/OSF.IO/SZQR8).

### Eligibility Criteria

Eligible countries were identified according to the World Bank’s definition of Sub-Saharan African region in June 2020, which included all of the following: Angola; Benin; Botswana; Burkina Faso; Burundi; Cabo Verde; Cameroon; Central African Republic; Chad; Comoros; Congo Dem. Rep.; Congo Rep.; Cote d’Ivoire; Equatorial Guinea; Eritrea; Eswatini; Ethiopia; Gabon; Gambia; Ghana; Guinea; Guinea-Bissau; Kenya; Lesotho; Liberia; Madagascar; Malawi; Mali; Mauritania; Mauritius; Mozambique; Namibia; Niger; Nigeria; Rwanda; Sao Tome and Principe; Senegal; Seychelles; Sierra Leone; Somalia; South Africa; South Sudan; Sudan; Tanzania; Togo; Uganda; Zambia; Zimbabwe (The World Bank Group, 2020). We considered all studies written in English language. Only studies published between January 2010 and June 2020 were included in order to accumulate prevalence data from January 2010 to December 2019. The aim of limiting the timeline of this review to a 10-year interval was to provide an updated, in-depth analysis of DENV epidemiology in Sub-Saharan Africa and to track the temporal changes in its prevalence in this region. Eligible articles also had to report a prevalence estimate of at least one of the following markers: DENV-specific IgG, IgM, or DENV RNA. Exclusion criteria included all of the following: (i) case reports, case series, reviews, editorial letters, conference proceedings and commentaries (ii) studies on non-native groups or travelers returning from the sub-Saharan region, (iii) studies reporting infection rates in non-human animals, (iv) entomologic and vector-related studies, and (v) studies for which sample collection was completed before 2010. No studies were excluded based on the quality of the used serologic assays or molecular techniques. This point was addressed under the assessment of risk of methodological bias during the quality assessment of included studies.

### Search Strategy

A comprehensive Literature search was commenced in June 2020. The targeted databases were MEDLINE/PubMed, ScienceDirect, Cochrane Library and Google Scholar. The databases were queried to search the “Titles and Abstracts” using the keywords “Dengue”, “Dengue Virus” and “DENV” in combination with the names of all eligible countries. The “publication date” filter was set at 10 years. The search strategy used in PubMed is demonstrated in **Supplementary Text 1**. This strategy was adapted to retrieve relevant articles from other databases. No articles were added from other sources in order to maintain the systematic setting of this review.

### Study Selection

The titles and abstracts of all retrieved citations were imported into Mendeley reference manager. Duplicates were removed using the same software. Two reviewers independently cross-examined all articles against a set of predetermined selection criteria. Any uncertainties were discussed by all authors, and a final decision was always reached by consensus. The full texts of all eligible studies were retrieved for further assessment. The corresponding author was contacted in the case of failure to retrieve the full text of any article. Failing to receive a response from an author within a waiting period of two weeks meant the exclusion of the corresponding article from this review.

### Data Extraction

Eligible articles were screened twice to insure an accurate extraction of data. The following information were extracted from each article: country of the study, date of publication, time of data collection, time-frame of study (e.g. during or outside outbreaks), location(s) of data collection, setting of the study, targeted sub-population(s), type(s) and manufacturer(s) of assay(s) used in the study, the reported infection marker(s), the sample size, the reported prevalence estimate(s) and the serotype(s) detected.

### Data Analysis

All articles were included in a quantitative meta-analysis to determine the overall and sub-group prevalence of DENV infection in Sub-Saharan Africa. If a study reported multiple prevalence estimates (e.g. in cases of multiple geographical areas, sub-populations or DENV infection markers), all estimates were entered separately in the meta-analysis. The prevalence estimate of each study was used as the effect estimate. The corresponding standard error (SE) for each study was calculated using the equation:

$$SE=\sqrt{\frac{p(1-p)}{n}}$$

where *p* is the reported prevalence estimate, and *n* is the sample size. Studies were weighted according to the prevalence effect size and the inverse of variance. Random-effects model was used to generate summary prevalence data (displayed on forest plots) with 95% confidence intervals. Heterogeneity analysis was done using the Inconsistency Index (*I* ^2^) as it’s known to be less influenced by the number of included studies. Funnel plots were created and visually-examined to investigate for possible publication bias. Sub-group analysis according to the country and time-frame of each study was performed in an effort to identify the cause(s) of significant statistical heterogeneity. Sub-group analysis according to the time of sample collection was performed to describe the changing patterns of prevalence of DENV infection markers (IgG, IgM and RNA) through the past decade. All statistical analysis was done using Microsoft Office Excel 2010 and Review Manager 5.3 (The Cochrane Collaboration) (Cochrane Collaboration, 2014).

### Quality Assessment

Two reviewers assessed the quality and risk of bias of all articles included in this review using a modified version of the Critical Appraisal Tool for prevalence studies designed by the Joanna Briggs Institute (JBI) and reported by Munn et al. in 2014 (Munn et al., 2014). This 10-question model was designed to assess the risk of confounding bias, selection bias, and bias related to measurement and data analysis. Each question was answered either with “yes”, “no”, “unclear” or “not/applicable”. A score was calculated as the number of questions answered with a “yes” for each study. According to this score, studies were categorized into three groups based on their risk of bias; high risk (a score of 0-3), intermediate risk (a score of 4-7), and low risk groups (a score of 8-10). A study was considered to represent its target population if the basic characteristics of its sample were found to mimic the basic characteristics of the targeted population and sub-populations. Random (probability) sampling was considered as the proper recruitment technique. The sample size had to be calculated by the authors of each study, as the calculation of a universally-adequate sample size was considered to be unreliable due to the wide variability in the basic characteristics of populations or sub-populations of different studies. If no sample size calculations were reported, “unclear” was given as an answer to this question. The following measurement techniques were determined to provide reliable prevalence estimates of DENV infection markers: Enzyme-Linked Immuno-Sorbent Assay (ELISA), ImmunoFluorescence Assay (IFA), Reverse-Transcriptase Polymerase Chain Reaction (RT-PCR) and Viral Neutralization Tests (VNT). Other techniques reported in some studies were subjects of discussion by the reviewing team and their reliability was determined only be consensus. Important confounding factors were considered to be properly addressed if the study findings were confirmed by PCR or an investigation for cross-reactivity against other arboviral infection markers was conducted (**Supplementary Table 2**).

## Results

### Study Characteristics

**Figure 1:**
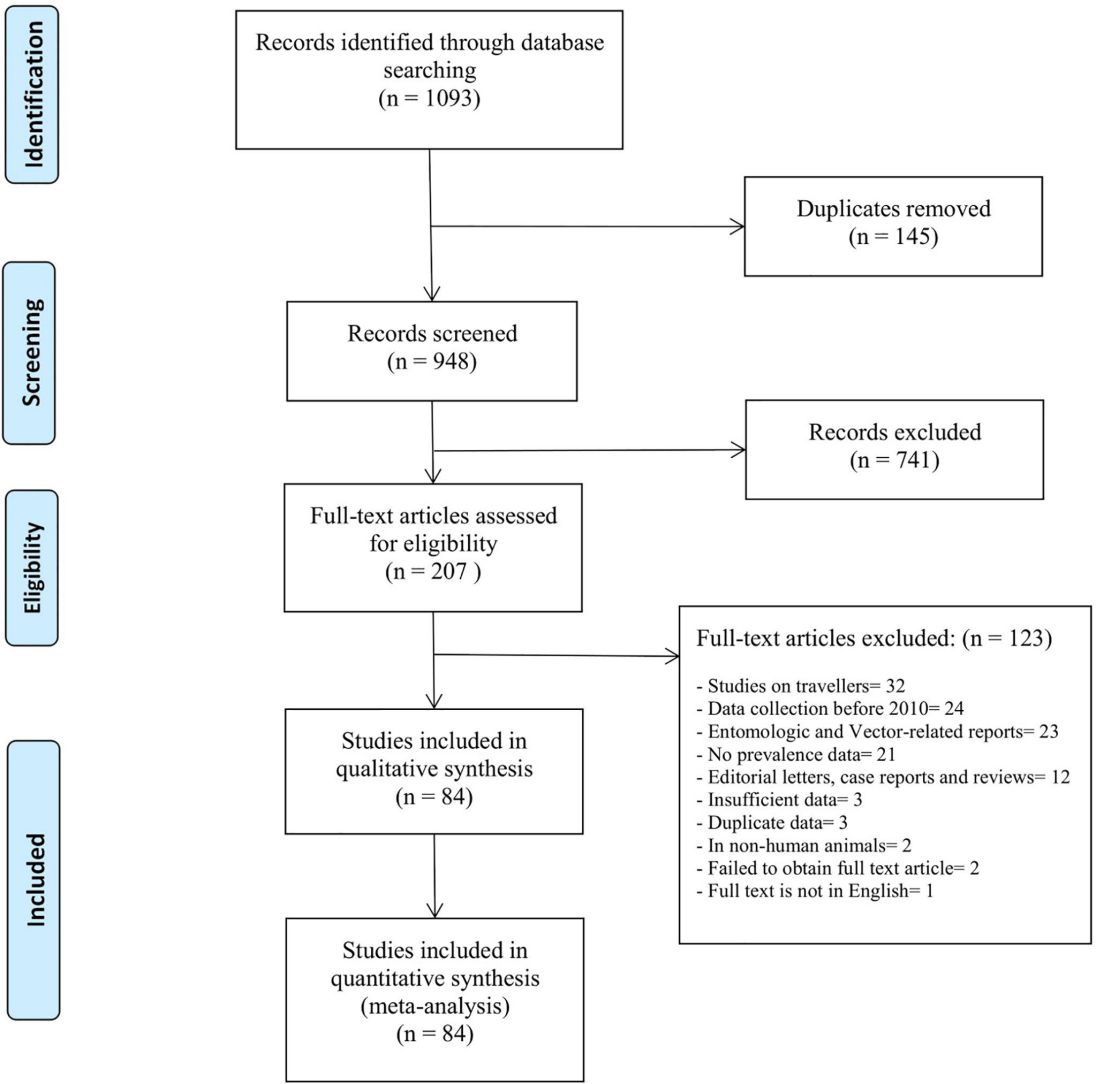
PRISMA flow-chart outlines the identification, screening and selection of eligible studies.

**Figure 2:**
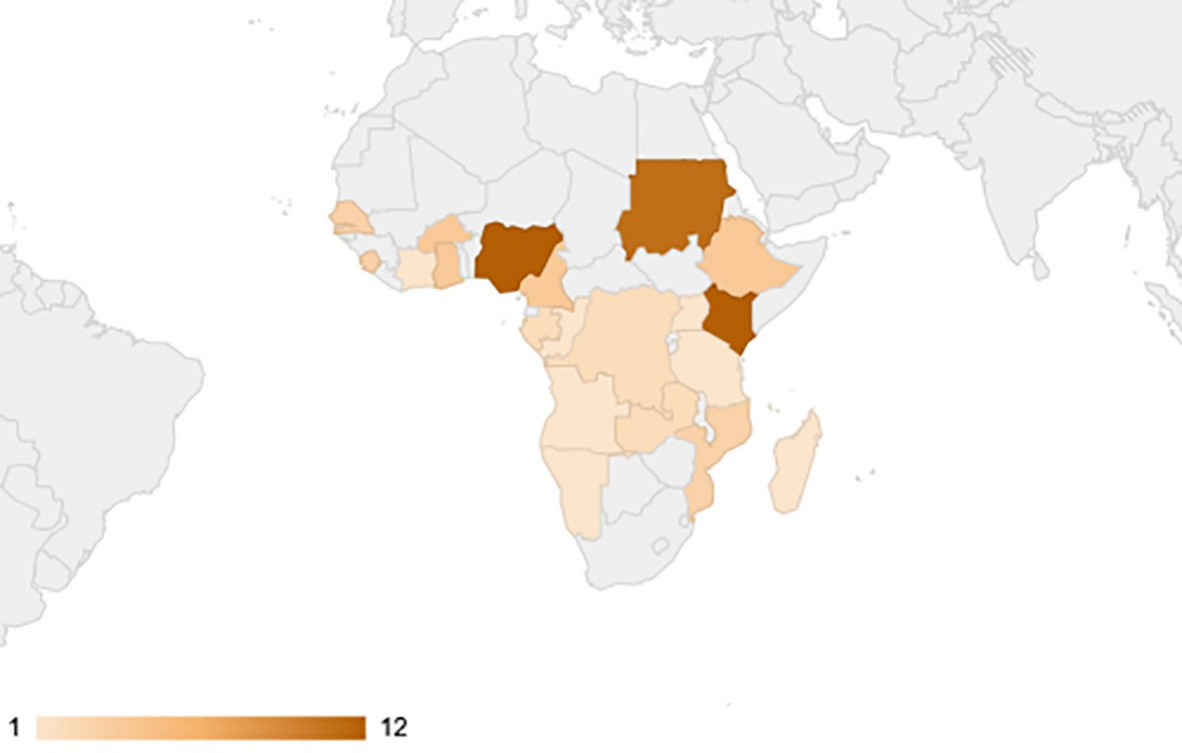
Choropleth map of Sub-Saharan African countries weighted according to the number of retrieved studies. No articles were found from countries highlighted in grey.

A total of 1093 studies were identified from the electronic literature search, of which 84 studies were included in this review (**Figure 1**). They reported prevalence estimates from a total of 21 countries in Sub-Saharan Africa, namely Angola (n=1), Burkina Faso (n=4), Cameroon (n=4), Comoros (n=1), Republic of the Congo (n=1), Cote d’Ivoire (n=1), Democratic Republic of the Congo (n=2), Ethiopia (n=4), Gabon (n=2), Ghana (n=4), Kenya (n=12), Madagascar (n=1), Mozambique (n=3), Namibia (n=1), Nigeria (n=12), Senegal (n=3), Sierra Leone (n=3), Sudan (n=11), Tanzania (n=11), Uganda (n=1) and Zambia (n=2) (**Figure 2**). Outlines of data extracted from all included studies are provided in **Supplementary Table 3**. A summary of the basic characteristics of included studies is provided in **Table 1**.

**Table 1:** Summary of the basic characteristics of included studies.

Study characteristic	n	%
Setting*		
Clinical	59	70.2%
Community-based	21	25%
others†	5	5.9%
Population		
Healthy individuals§	24	28.6%
Suspected Dengue virus illness patients	10	11.9%
Acute febrile illness patients	46	54.8%
Others‡	4	4.8%
Diagnostic assays		
ELISA IgM	41	48.8%
ELISA IgG	44	52.4%
IFA	3	3.6%
ICT	4	4.8%
RDT	7	8.3%
RT-PCR	35	41.7%
RT-RPA	1	1.2%
Multiplex nested PCR	1	1.2%
Time-frame¶		
During an outbreak	15	17.9%
Outside outbreaks	70	83.3%
Quality assessment		
Low risk of bias	27	32.1%
Intermediate risk of bias	57	67.9%
High risk of bias	0	0%
DENV serotypes**		
DENV-1	12	14.2%
DENV-2	16	19%
DENV-3	16	19%
DENV-4	3	3.5%

*One study described both hospital and community-based settings.

†Others include blood donation clinics (n=3), antenatal care centers (n=1) and a university (n=1).

‡Others include suspected cases of HIV(n=1), measles (n=1), Ebola (n=1), malaria (n=1), chikungunya (n=1).

§Healthy individuals include volunteer blood donors (n =3), pregnant women (n=1) and university students (n=1).

¶One study reported prevalence estimates both during and outside an outbreak.

**DENV serotype was specified if the serological assay was declared to be specific for a single serotype, or in the case of serotype confirmation by polymerase chain reaction, virus neutralization test, isolation of the virus and sequencing of viral nucleic acid.

ELISA, enzyme-linked immuno-sorbent assay; ICT, immunochromatography assay; IFA, immunofluorescence assay; RDT, rapid diagnostic test; RT-PCR, reverse transcriptase polymerase chain reaction; RT-RPA, reverse transcriptase recombinase polymerase amplification.

### Prevalence of DENV Infection in Sub-Saharan Africa

**Figure 3:**
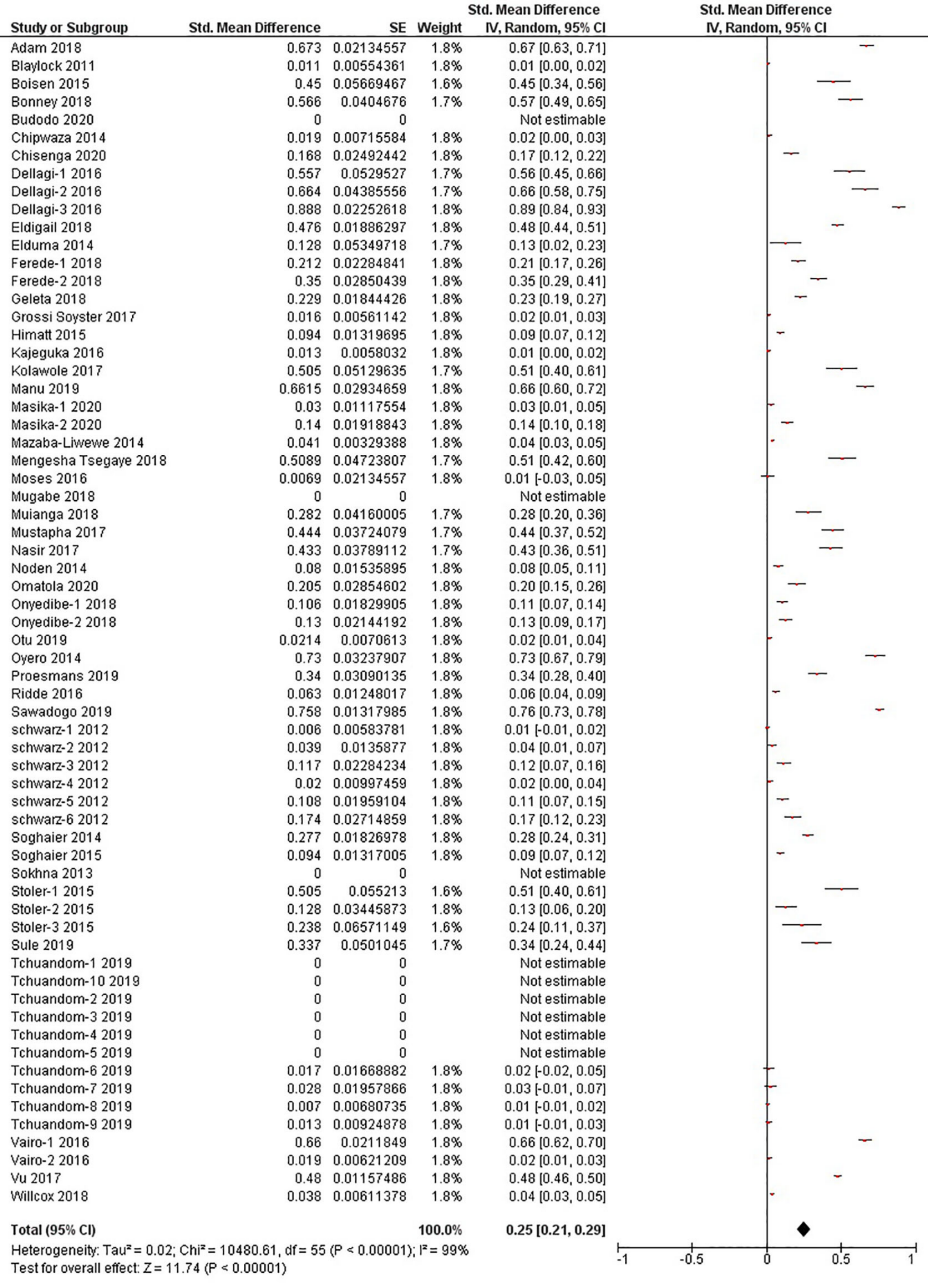
The pooled sero-prevalence of Dengue Virus Immunoglobulin G (IgG) in Sub-Saharan African population between 2010-2020.

The quantitative meta-analysis evaluated data from all included articles, which provided a total of 136 prevalence estimates. Meta-analysis was performed separately for each of the DENV infection markers (IgG, IgM, and RNA). Random effects analysis estimated a pooled IgG prevalence of 25% (95% CI: 21-29%), a pooled IgM prevalence of 10% (95% CI: 9-11%) and a pooled DENV RNA prevalence of 14% (95% CI: 12-16%) (**Figures 3** **–** **5**). Significantly high levels of heterogeneity between studies were found in all three meta-analyses (*I* ^2^ = 99%, 98% and 99%, respectively). Evidence of possible publication bias was found in all three meta-analyses by visual examination of the resulting funnel plots (i.e. revealing asymmetrical distributions of studies on either side of the overall pooled prevalence estimates) (**Supplementary Figures 1–3**).

**Figure 4:**
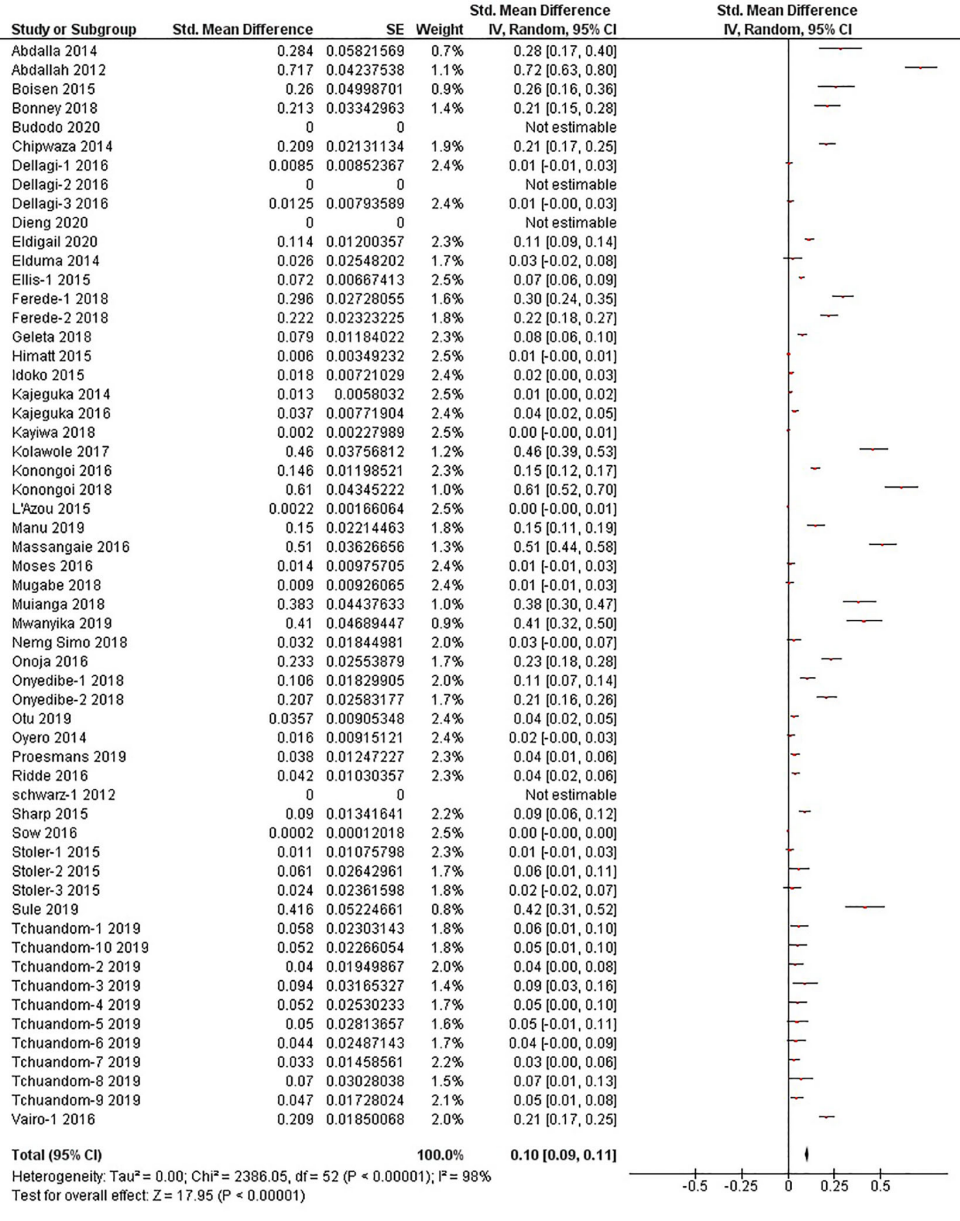
The pooled sero-prevalence of Dengue Virus Immunoglobulin M (IgM) in Sub-Saharan African population between 2010-2020.

**Figure 5:**
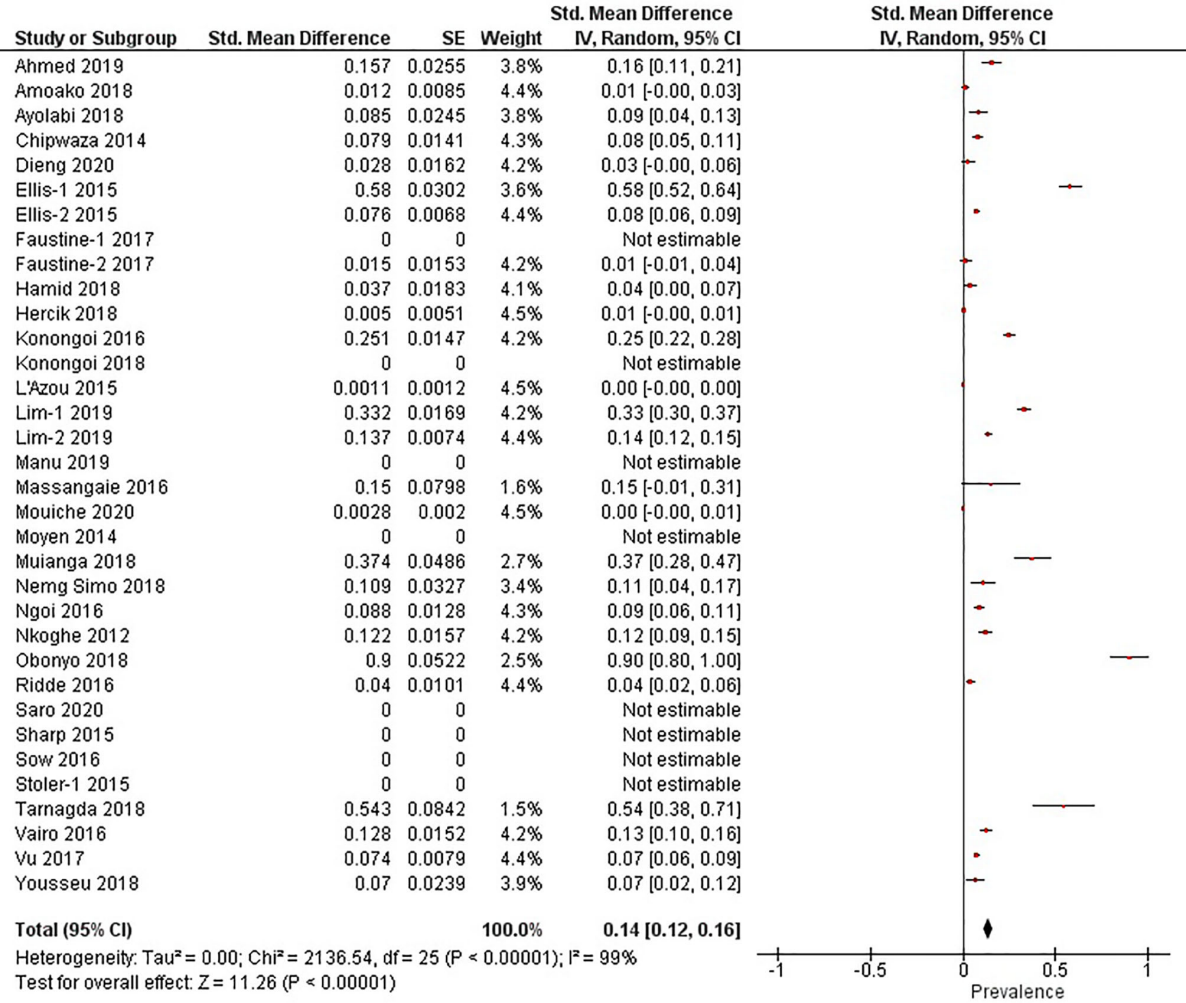
The pooled prevalence of Dengue Virus RNA in Sub-Saharan African population between 2010-2020.

### Sub-Group Analysis of DENV Infection

**Figure 6:**
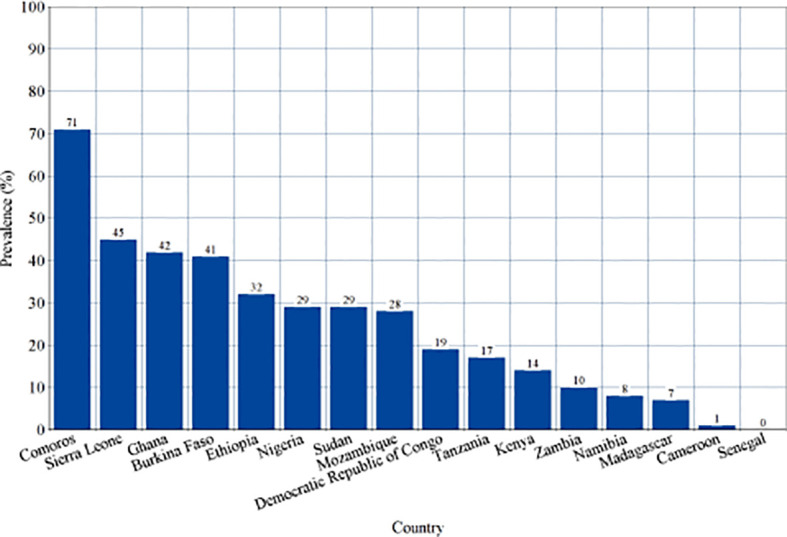
Sero-prevalence of Dengue-specific Immunoglobulin G (IgG) in countries of Sub-Saharan Africa. This is a visual representation of subgroup analysis of studies reporting the sero-prevalence of Dengue virus IgG in Sub-Saharan African population between 2010-2020 according to the country of study.

Subgroup analysis of studies reporting the sero-prevalence of IgG antibodies was done according to the country of study (**Supplementary Figure 4**). Significantly high levels of heterogeneity were found in all countries (*I* ^2^ ranging between 93-100%) with a significant difference between them (p<0.00001, *I* ^2 ^= 94.1%). Countries such as Comoros, Sierra Leone, Ghana and Burkina Faso were found to harbor the highest levels of pooled IgG sero-prevalence (71%, 45%, 42% and 41%, respectively) (**Figure 6**). Alternatively, studies reporting the sero-prevalence of IgM antibodies were sub-grouped according to the time-frame of the study (i.e. during or outside outbreaks). Analysis also revealed a significantly high level of heterogeneity in both subgroups (*I* ^2 ^= 98% in both of them) with a significant difference between them (p<0.00001, *I* ^2 ^= 88.9%) (**Supplementary Figure 5**). Studies reporting the prevalence of DENV RNA were also sub-grouped according to the time-frame of the study, and were also found to have a high level of heterogeneity (*I* ^2^ = 99% in both of them) and a significant difference between the two subgroups (p<0.00001, *I* ^2 ^= 95.9%) (**Supplementary Figures 5, 6**).

### Temporal Trends in Prevalence of Infection Markers

**Figure 7:**
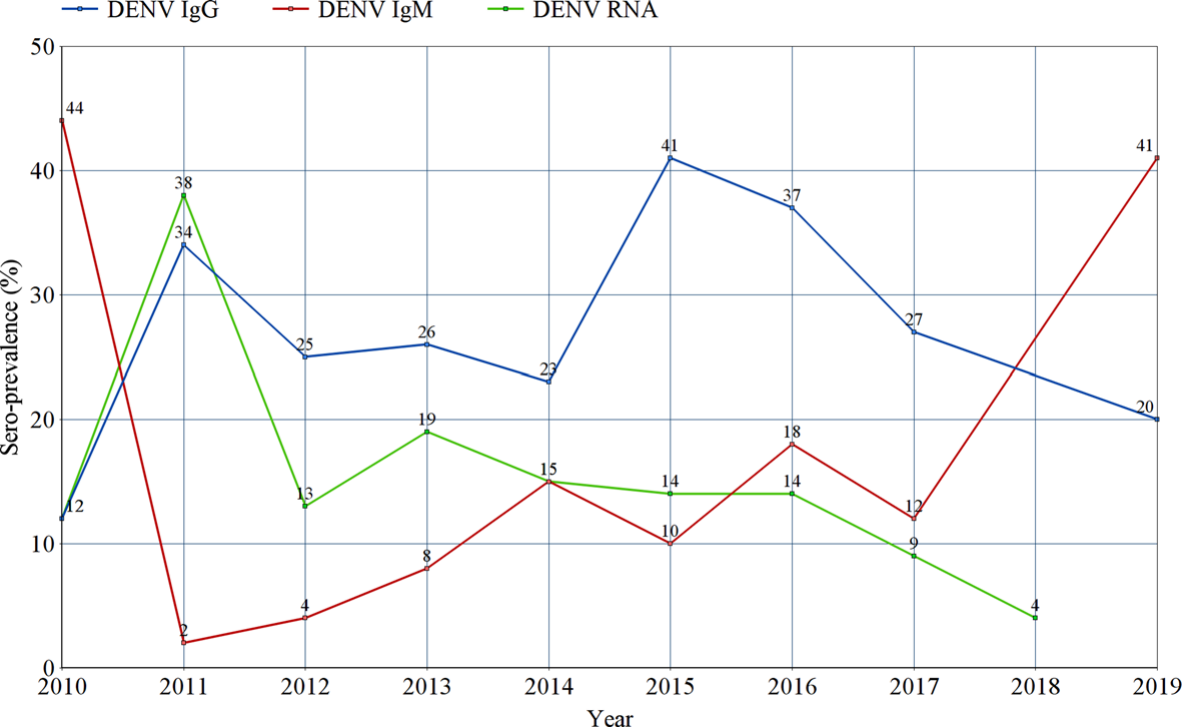
Tracking the prevalence estimates of Dengue-specific IgG, IgM, and Dengue virus RNA in Sub-Saharan Africa between 2010 and 2019. Each point represents the random effects pooled prevalence of the specified infection marker from studies reporting sample collection in the corresponding year. No prevalence estimates of IgG or IgM were reported in 2018, nor for RNA in 2019.

All studies were sub-grouped according to the year(s) of sample collection. Pooled prevalence estimates of IgG, IgM and RNA were calculated for every year since 2010 and all estimates were entered into a line graph to track the changing prevalence patterns of DENV infection markers in Sub-Saharan Africa through the past ten years (**Figure 7** and **Supplementary Figures 7–9**).

### Prevalence According to the Clinical Classification of Patients

**Figure 8:**
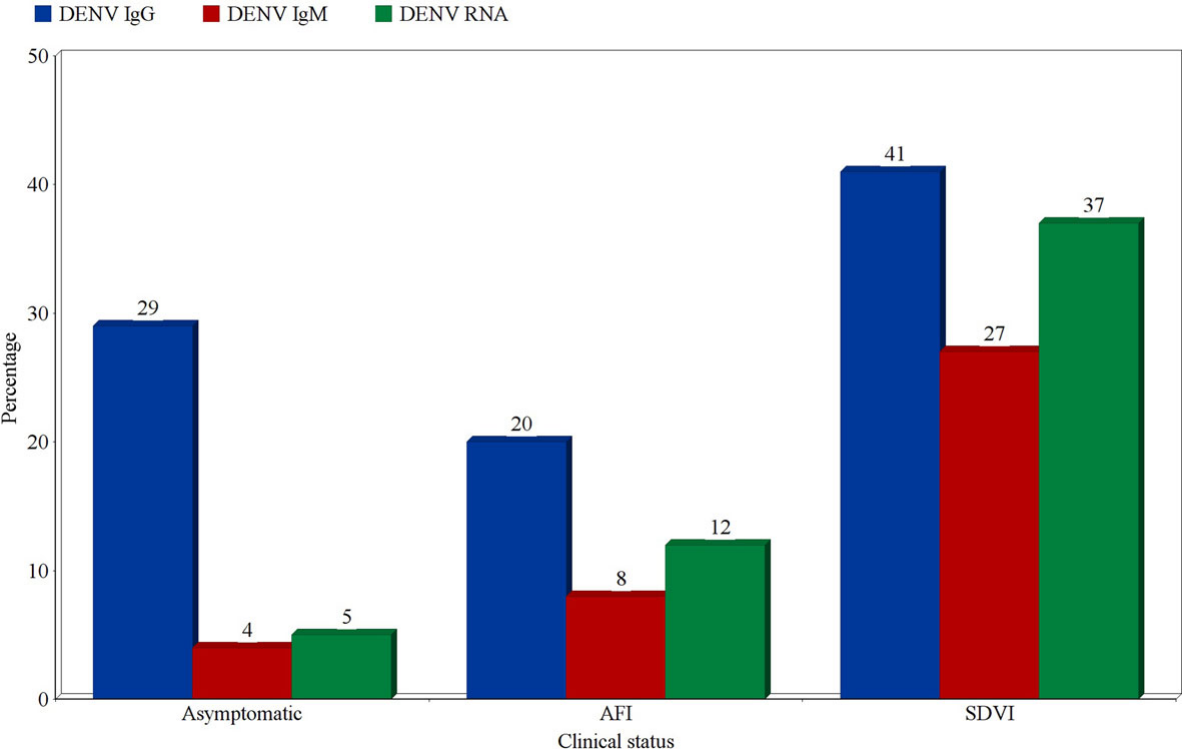
Prevalence estimates of Dengue-specific IgG, IgM, and Dengue virus RNA in asymptomatic participants, patients with acute febrile illness (AFI), and patients with suspected Dengue virus infection (SDVI). Each estimate represents the random effects pooled prevalence of the specified infection marker from studies focusing on any of these clinically-defined groups.

Prevalence estimates of DENV infection markers were calculated for three categories of participants; asymptomatic participants, patients presenting with an acute febrile illness (AFI), and patients with suspected DENV infections (SDVI) according to the Centers for Disease Control and Prevention’s (CDC) clinical case definition. The highest prevalence estimates of probable and confirmed cases were found in the SDVI group, as 27% were positive for DENV-IgM (95% CI:14-40%, *I* ^2 ^= 97%), and 37% were positive for DENV-RNA (95% CI:10-64%, *I* ^2 ^= 99%). Moreover, 8% (95% CI:7-9%, *I* ^2 ^= 97%) and 12% (95% CI:8-15%, *I* ^2 ^= 99%) of patients with an AFI were found to be positive for DENV-IgM and RNA, respectively. Regarding the asymptomatic participants, 4% (95% CI:1-7%, *I* ^2 ^= 95%) were IgM-positive and 5% (95% CI:0-11%, *I* ^2^ = 92%) were RNA-positive. Other estimates can be seen in **Figure 8**.

## Discussion

DENV infections are known to follow an epidemiological pattern that generally resembles those of other re-emerging infections, including other arboviral diseases. This pattern results in an apparent fluctuation both in the clinical and serological prevalence estimates of such diseases, due to the spiking increase commonly recorded during outbreaks, and the rapid drop that follows afterwards. Hence, the epidemiology of emerging and re-emerging infectious diseases is better described over a prolonged period of time, as a point estimate can rarely reflect the true burden of these conditions in a designated area. This systematic review and meta-analysis of the prevalence of DENV infection in Sub-Saharan Africa depicted high prevalence estimates in the past ten years, with a significantly high level of heterogeneity that varies according to country and time-frame of studies, and also according to the infection marker of interest.

According to the findings of this review, 10-14% of the Sub-Saharan African population has developed an acute DENV infection in the past ten years, with 25% of this population having a history of exposure to DENV at some point during their lives, evidenced by their sero-positivity to DENV-specific IgG. The fact that these prevalence estimates were calculated through meta-analysis of individual primary studies and not direct products of proper national or regional surveillance systems raises the possibility of a significant underestimation of the true burden of DENV infection in Sub-Saharan Africa. The high prevalence of DENV in this region implies either the absence or failure of vector control programs; the most important disease control strategy when DENV infection is concerned. Some countries, such as Cuba, successfully developed an integrated system to control and prevent the transmission of DENV (Bisset et al., 2011). However, countries in Sub-Saharan Africa are generally considered to lack the necessary infrastructure and resources for a successful implementation and maintenance of such programs. Additionally, the emergence of some forms of resistance to insecticides among vectors of DENV, and the increasing rate of urbanization and human mobility in the region markedly exacerbate the challenge of controlling the spread of those vectors (Vontas et al., 2012; Liu, 2015).

We conducted a subgroup meta-analysis of all included studies according to the year(s) of sample collection in an effort to track the changes in prevalence of all DENV infection markers (IgG, IgM and RNA) through the past decade. In **Figure 7**, the re-emerging pattern of DENV infection in Sub-Saharan Africa is easily observed. Regarding IgM and RNA, their prevalence patterns can be described as series of a spiking increase followed by a rapid drop in prevalence. This is probably due to the short-lived positivity to IgM and RNA following an acute infection with DENV. The spikes in IgM and RNA levels seen between 2010 and 2011 can be explained by the concurrent outbreaks in Gabon, Kenya and Sudan (Ngoi et al., 2016). Similar spikes can be seen between 2013 and 2014, coinciding with the outbreaks taking place in Angola, Burkina Faso, Kenya, Mozambique and Tanzania (Vairo et al., 2014; Ridde et al., 2016). Finally, a solitary spike in IgM can be observed in 2016, without a recorded increase in RNA prevalence. This spike marks two outbreaks taking place in Burkina Faso and Sudan (Sawadogo et al., 2020). On the other hand, the sero-prevalence of DENV-specific IgG is seen as series of a spiking increase followed by a slow, gradual decline in sero-prevalence over the following years, but it does not seem to return to baseline. Two spikes in IgG sero-prevalence can be seen in 2011 and 2015, shortly after the two clusters of outbreaks described in 2010 (Sudan, Gabon and Kenya) and 2014 (Angola, Kenya, Burkina Faso, Mozambique and Tanzania), respectively. These spikes are attributed to the increased exposure of the Sub-Saharan population to DENV during these outbreaks. The slow decline in IgG sero-prevalence that follows can be explained by population turnover and death of sero-positive individuals, as IgG sero-positivity is expected to be maintained for life.

We also found that the pooled prevalence estimates of DENV-specific IgM and RNA can record a three-to-five-folds increase during outbreaks in this region. Taking into account that we also provide evidence for the circulation of all four DENV serotypes in Sub-Saharan Africa, this population has a very high risk of secondary infections with heterologous DENV serotypes. As a result, future DENV outbreaks in this region are expected to record very high incidences of severe DENV infections, such as Dengue Hemorrhagic Fever (DHF) and Dengue Shock Syndrome (DSS), due to the phenomenon of Antibody-Dependent Enhancement (ADE). This highlights the importance of developing specialized surveillance systems in the region to monitor the incidence and prevalence of DENV along with other re-emerging infectious diseases, and more importantly, to identify any changes in the predominant DENV serotypes in all countries and subregions in order to predict the catastrophic occurrence of such outbreaks.

In its 2021-2030 roadmap against Neglected Tropical Diseases (NTDs), the World Health Organization (WHO) prioritized a group of 20 NTDs, including dengue fever, that have a devastating impact on low-resource communities, imposing a global human, economic and social burden. Achieving the targets of this ambitious strategy will require a global collaboration of all partners to *“accelerate programme actions against NTDs, including interventions to reduce incidence, prevalence, morbidity, disability and death”* (World Health Organization, 2020). This greatly emphasizes the need for a thorough, in-depth understanding of the epidemiology of these diseases especially in regions of the world such as the Sub-Saharan Africa, which has a very high burden of these conditions, and a poor implementation of surveillance systems and control programs. On the other hand, the expected DENV-specific target highlighted by the WHO in this roadmap is to achieve a better control of DENV, by reducing its case fatality to 0% by 2030 (World Health Organization, 2020). The placement of DENV among this group of diseases, which are only “targeted for control”, implies that the WHO recognizes our current understanding of this virus as inadequate to achieve neither its elimination as a public health problem nor its eradication anytime in the foreseen future.

The findings of this study provide a valuable insight into the contemporaneous epidemiology of DENV infections in this region. These results can inform future planning and implementation of any interventional or preventive strategies against DENV in this region; e.g. informing the utility of a vaccine. Furthermore, since no specific treatment (e.g. anti-viral agents) has been developed against DENV, understanding its epidemiology among the different socio-demographic groups, and distinct clinical categories is vital to understand the burden of DENV infections to regional healthcare systems. However, this systematic review and meta-analysis has its limitations; the most important of which is the issue of cross-reactivity in the diagnostic serologic techniques. There is evidence of cross-reactivity of DENV ELISA assays with other flaviviruses; such as West Nile virus, Zika virus and Yellow fever virus, as well as other non-flavivirus infections such as malaria (Lam et al., 2000; Maeki et al., 2011; Papa et al., 2011; Zaidi et al., 2020). Sub-Saharan Africa is known to harbor a high burden of these infections. As a result, confirmatory tests such as real-time (quantitative) polymerase chain reaction (qPCR) and plaque reduction neutralization test (PRNT) are crucial to confirm the findings of these serologic assays. Nevertheless, ELISA remains the most commonly used test in the surveillance of DENV and other flaviviral infections, especially in this region, mainly due to its low-resource nature, but also due to the advanced lab settings needed for proper application of other techniques. Other limitations can also be seen, as we were unable to retrieve any prevalence reports from some countries in this region, possibly affecting the generalizability of our results. Additionally, these results were found to have a high level of heterogeneity that could not be explained by subgroup differences according to country of the study or its time-frame in relation to outbreaks. This indicates an even more profound basis to this heterogeneity, such as the difference in population characteristics and the methodological heterogeneity generated by the use of different serologic techniques (such as ELISA, IFA and ICT) to estimate DENV IgG and IgM sero-prevalence, and target sequence heterogeneity and the use of different thresholds for positivity in the case of RNA prevalence. Moreover, these results should be carefully interpreted due to the evidence of possible publication bias reported in this meta-analysis. However, funnel plot asymmetry may also be a result of the significant clinical or methodological heterogeneity noted between the studies in this meta-analysis and not a mere reflection of the risk of publication bias (Duval and Weinhandl, 2011). Thus, the potential impact of publication bias should be re-assessed using more computationally-demanding techniques such as the trim and fill method or the selection model approach.

## Conclusion

Sub-Saharan Africa was found to have a high prevalence of DENV infections. Due to the absence of an effective vaccine or anti-viral treatment, more efforts should be done to control its vectors, to establish comprehensive surveillance systems and to closely monitor any significant changes in its epidemiology in this region.

## Author Contributions

Conceptualization: KEl and KEn. Methodology: KEl, KEn, and AE. Formal analysis: KEl Writing - original draft preparation: KEl and KEn. Writing - review and editing: AE and IE. Visualization: KEl Supervision, AE and IE. All authors contributed to the article and approved the submitted version.

## Conflict of Interest

The authors declare that the research was conducted in the absence of any commercial or financial relationships that could be construed as a potential conflict of interest.
