# Supplementary material for: Dengue Virus Infection in Sub-Saharan Africa Between 2010 and 2020: A Systematic Review and Meta-Analysis
Source: Front Cell Infect Microbiol. 2021 May 25;11:678945. doi: 10.3389/fcimb.2021.678945 (PMC8186319; doi:10.3389/fcimb.2021.678945)
Supplement: Supplementary file 1 [file DataSheet_1.docx]

**Search strategy in PubMed**

**Query Box:**

(("dengue"[Title/Abstract]) OR ("dengue virus"[Title/Abstract]) OR ("DENV"[Title/Abstract])) AND (("Angola"[Title/Abstract]) OR ("Benin"[Title/Abstract]) OR ("Botswana"[Title/Abstract]) OR ("Burkina Faso"[Title/Abstract]) OR ("Burundi"[Title/Abstract]) OR ("Cabo Verde"[Title/Abstract]) OR ("Cameroon"[Title/Abstract]) OR ("Central African Republic"[Title/Abstract]) OR ("Chad"[Title/Abstract]) OR ("Comoros"[Title/Abstract]) OR ("Congo"[Title/Abstract]) OR ("Cote d'Ivoire"[Title/Abstract]) OR ("Eritrea"[Title/Abstract]) OR ("Eswatini"[Title/Abstract]) OR ("Ethiopia"[Title/Abstract]) OR ("Gabon"[Title/Abstract]) OR ("Gambia"[Title/Abstract]) OR ("Ghana"[Title/Abstract]) OR ("Bissau"[Title/Abstract]) OR ("Guinea"[Title/Abstract]) OR ("Kenya"[Title/Abstract]) OR ("Lesotho"[Title/Abstract]) OR ("Liberia"[Title/Abstract]) OR ("Madagascar"[Title/Abstract]) OR ("Malawi"[Title/Abstract]) OR ("Mali"[Title/Abstract]) OR ("Mauritania"[Title/Abstract]) OR ("Mauritius"[Title/Abstract]) OR ("Mozambique"[Title/Abstract]) OR ("Namibia"[Title/Abstract]) OR ("Niger"[Title/Abstract]) OR ("Nigeria"[Title/Abstract]) OR ("Rwanda"[Title/Abstract]) OR ("Principe"[Title/Abstract]) OR ("Sao Tome"[Title/Abstract]) OR ("Senegal"[Title/Abstract]) OR ("Seychelles"[Title/Abstract]) OR ("Sierra Leone"[Title/Abstract]) OR ("Somalia"[Title/Abstract]) OR ("South Africa"[Title/Abstract]) OR ("South Sudan"[Title/Abstract]) OR ("Sudan"[Title/Abstract]) OR ("Tanzania"[Title/Abstract]) OR ("Togo"[Title/Abstract]) OR ("Uganda"[Title/Abstract]) OR ("Zambia"[Title/Abstract]) OR ("Zimbabwe"[Title/Abstract]) )

**Filters:**

Publication date= 10 years.
