# Supplementary material for: Dengue Virus Infection in Sub-Saharan Africa Between 2010 and 2020: A Systematic Review and Meta-Analysis
Source: Front Cell Infect Microbiol. 2021 May 25;11:678945. doi: 10.3389/fcimb.2021.678945 (PMC8186319; doi:10.3389/fcimb.2021.678945)
Supplement: Supplementary file 11 [file Table_2.docx]

Supplementary table 2. Quality assessment (critical appraisal) of included studies using the Joanna Briggs Institute (JBI) critical appraisal tool for prevalence studies.

|  | *Was the sample representative of the target population?** | *Were study participants recruited in an appropriate way?* **^†^** | *Was the sample size adequate?***^‡^** | *Were the study subjects and setting described in detail?* | *Is the data analysis conducted with sufficient coverage of the identified sample?* | *Were objective, standard criteria used for measurement of the condition?* | *Was the condition measured reliably?***^§^** | *Was there appropriate statistical analysis?* | *Are all important confounding factors/ subgroups/ differences identified and accounted for?***^¶^** | *Were subpopulations identified using objective criteria?* | Total  Score |
| --- | --- | --- | --- | --- | --- | --- | --- | --- | --- | --- | --- |
| Abdalla  [60] | yes | yes | u/c | yes | u/c | yes | yes | yes | yes | yes | 7 |
| Abdallah  [61] | u/c | yes | u/c | yes | yes | yes | yes | yes | yes | yes | 8 |
| Abe  [19] | yes | yes | yes | yes | yes | yes | yes | yes | yes | yes | 10 |
| Adam  [62] | yes | yes | yes | yes | yes | yes | yes | yes | yes | yes | 10 |
| Ahmed  [63] | u/c | yes | u/c | yes | no | yes | yes | yes | yes | yes | 7 |
| Amoako  [31] | Yes | Yes | u/c | yes | yes | yes | yes | yes | yes | u/c | 8 |
| Ayolabi  [42] | u/c | yes | u/c | yes | yes | yes | yes | yes | yes | yes | 8 |
| Blaylock  [25] | u/c | u/c | yes | yes | yes | yes | yes | yes | u/c | no | 6 |
| Boillat-Blanco  [71] | yes | yes | yes | yes | yes | yes | no | yes | yes | yes | 9 |
| Boisen  [57] | u/c | yes | u/c | yes | u/c | yes | yes | yes | yes | yes | 7 |
| Bonney  [22] | u/c | u/c | u/c | yes | yes | yes | yes | yes | u/c | yes | 6 |
| Budodo  [72] | u/c | u/c | u/c | yes | yes | yes | yes | yes | u/c | yes | 6 |
| Chepkorir  [26] | yes | yes | yes | yes | yes | yes | yes | yes | yes | yes | 10 |
| Chipwaza  [73] | yes | u/c | u/c | yes | yes | yes | yes | yes | yes | yes | 8 |
| Chisenga  [83] | u/c | u/c | u/c | yes | yes | yes | yes | yes | yes | yes | 7 |
| Dariano  [69] | u/c | u/c | yes | yes | yes | yes | yes | yes | u/c | yes | 7 |
| de Araujo Lobo  [59] | u/c | u/c | u/c | yes | yes | yes | yes | yes | yes | no | 6 |
| Degife  [15] | u/c | u/c | u/c | yes | u/c | yes | no | yes | yes | yes | 5 |
| Dellagi  [10] | u/c | u/c | yes | yes | yes | yes | yes | yes | u/c | yes | 7 |
| Dieng  [54] | no | u/c | u/c | yes | yes | yes | yes | yes | yes | no | 6 |
| Eldigail  [64] | yes | yes | yes | yes | yes | yes | yes | yes | u/c | u/c | 8 |
| Eldigail  [65] | yes | yes | yes | yes | yes | yes | yes | yes | u/c | yes | 9 |
| Elduma  [66] | no | no | no | yes | yes | yes | yes | yes | u/c | u/c | 5 |
| Ellis  [27] | yes | yes | yes | yes | yes | yes | yes | yes | yes | yes | 10 |
| Faustine  [74] | u/c | u/c | u/c | yes | yes | yes | yes | yes | u/c | yes | 6 |
| Ferede  [16] | yes | u/c | yes | yes | yes | yes | yes | yes | u/c | yes | 8 |
| Geleta  [17] | u/c | yes | yes | yes | yes | yes | yes | yes | yes | yes | 9 |
| Grossi Soyster  [28] | yes | yes | u/c | yes | yes | yes | yes | yes | u/c | u/c | 7 |
| Hamid  [67] | u/c | u/c | u/c | yes | yes | yes | yes | yes | u/c | u/c | 5 |
| Hercik  [75] | u/c | u/c | u/c | yes | yes | yes | yes | yes | yes | yes | 7 |
| Himatt  [68] | yes | yes | no | yes | yes | yes | yes | yes | u/c | yes | 8 |
| Idoko  [43] | u/c | u/c | yes | yes | yes | yes | yes | yes | u/c | u/c | 6 |
| Inziani  [29] | u/c | u/c | u/c | yes | yes | yes | yes | yes | yes | yes | 7 |
| Kajeguka  [76] | yes | u/c | yes | yes | yes | yes | yes | yes | u/c | yes | 8 |
| Kajeguka  [77] | u/c | u/c | u/c | yes | yes | yes | yes | yes | u/c | yes | 6 |
| Kayiwa  [82] | u/c | u/c | u/c | yes | yes | yes | yes | yes | u/c | u/c | 5 |
| Kolawole  [44] | u/c | u/c | u/c | yes | yes | yes | yes | yes | u/c | N/A | 5 |
| Konongoi  [30] | u/c | u/c | u/c | yes | yes | yes | yes | yes | u/c | yes | 6 |
| Konongoi  [31] | u/c | u/c | u/c | yes | yes | yes | yes | yes | yes | yes | 7 |
| L'Azou  [12] | u/c | yes | u/c | yes | yes | yes | yes | yes | yes | N/A | 7 |
| Lim  [2] | yes | u/c | u/c | yes | yes | yes | yes | yes | yes | yes | 7 |
| Manu  [23] | u/c | u/c | u/c | yes | yes | yes | yes | yes | u/c | N/A | 5 |
| Masika  [32] | u/c | u/c | u/c | yes | yes | yes | yes | no | u/c | no | 4 |
| Massangaie  [38] | u/c | u/c | u/c | yes | yes | yes | no | yes | u/c | yes | 6 |
| Mazaba-Liwewe  [84] | yes | yes | yes | yes | yes | yes | yes | yes | yes | yes | 10 |
| Mengesha Tsegaye  [18] | yes | yes | yes | yes | yes | yes | u/c | yes | u/c | yes | 8 |
| Moses  [45] | u/c | u/c | u/c | yes | yes | yes | no | yes | u/c | yes | 5 |
| Mouiche  [6] | u/c | u/c | u/c | yes | yes | yes | yes | yes | yes | yes | 7 |
| Moyen  [11] | yes | u/c | u/c | yes | yes | yes | yes | yes | yes | yes | 8 |
| Mugabe  [39] | u/c | u/c | u/c | yes | yes | yes | yes | yes | u/c | yes | 6 |
| Muianga  [40] | u/c | u/c | u/c | yes | yes | yes | yes | yes | u/c | yes | 6 |
| Mustapha  [46] | u/c | u/c | yes | yes | yes | yes | yes | yes | u/c | yes | 7 |
| Mwanyika  [78] | u/c | u/c | u/c | yes | yes | yes | no | yes | u/c | u/c | 4 |
| Nasir  [47] | u/c | u/c | yes | yes | yes | yes | yes | yes | u/c | yes | 7 |
| Nemg Simo  [7] | u/c | u/c | u/c | yes | yes | yes | yes | yes | u/c | yes | 6 |
| Ngoi  [33] | yes | u/c | u/c | yes | yes | yes | yes | yes | yes | yes | 8 |
| Nkoghe  [20] | u/c | u/c | u/c | yes | yes | yes | yes | yes | yes | yes | 7 |
| Noden  [41] | u/c | u/c | u/c | yes | yes | yes | yes | yes | no | yes | 6 |
| Obonyo  [34] | u/c | u/c | u/c | yes | yes | yes | yes | yes | yes | yes | 7 |
| Omatola  [48] | yes | yes | u/c | yes | yes | no | no | yes | no | yes | 6 |
| Onoja  [50] | u/c | u/c | u/c | yes | yes | yes | yes | yes | yes | yes | 7 |
| Onyedibe  [49] | u/c | u/c | u/c | yes | yes | yes | yes | yes | u/c | yes | 6 |
| Otu  [51] | yes | yes | yes | yes | yes | yes | yes | yes | u/c | yes | 9 |
| Oyero  [52] | u/c | u/c | no | yes | yes | yes | yes | yes | no | yes | 6 |
| Proesmans  [14] | no | u/c | no | yes | yes | yes | yes | yes | yes | yes | 7 |
| Ridde  [3] | u/c | u/c | u/c | yes | no | yes | no | yes | no | yes | 4 |
| Saro  [79] | u/c | u/c | u/c | yes | yes | yes | yes | yes | yes | N/A | 6 |
| Sawadogo  [4] | u/c | u/c | no | yes | yes | yes | yes | yes | u/c | yes | 6 |
| Schwarz  [37] | no | u/c | u/c | yes | yes | yes | yes | yes | u/c | yes | 6 |
| Sharp  [1] | yes | yes | u/c | yes | yes | yes | yes | yes | u/c | yes | 8 |
| Soghaier  [69] | yes | yes | yes | yes | yes | yes | yes | yes | u/c | yes | 9 |
| Soghaier  [70] | yes | yes | no | yes | yes | yes | yes | yes | u/c | yes | 8 |
| Sokhna  [55] | u/c | u/c | u/c | yes | yes | yes | no | yes | u/c | u/c | 4 |
| Sow  [56] | u/c | u/c | u/c | yes | yes | yes | yes | yes | u/c | no | 5 |
| Stoler  [24] | u/c | u/c | u/c | yes | yes | yes | yes | yes | u/c | yes | 6 |
| Sule  [53] | no | yes | u/c | yes | yes | yes | yes | yes | u/c | yes | 7 |
| Tarnagda  [5] | u/c | u/c | u/c | yes | yes | yes | no | yes | u/c | yes | 5 |
| Tchuandom  [8] | yes | yes | yes | yes | yes | yes | yes | yes | u/c | yes | 9 |
| Vairo  [80] | no | yes | u/c | yes | yes | yes | yes | yes | no | yes | 7 |
| Vairo  [81] | u/c | yes | u/c | yes | yes | yes | no | yes | yes | yes | 7 |
| Vu  [35] | yes | yes | u/c | yes | yes | yes | yes | yes | yes | yes | 9 |
| Vu  [36] | u/c | yes | u/c | yes | yes | yes | yes | yes | yes | yes | 8 |
| Willcox  [14] | yes | yes | u/c | yes | yes | yes | yes | yes | yes | yes | 9 |
| Yousseu  [9] | u/c | u/c | u/c | yes | yes | yes | yes | yes | yes | yes | 7 |

* A study was considered to represent its target population if the basic sample characteristics of this study were found to mimic the basic characteristics of the targeted population and sub-populations.

**^†^** Random (probability) sampling was considered as the proper recruitment technique.

**^‡^** The sample size had to be calculated by the authors of each study, as the calculation of a universally-adequate sample size was considered to be unreliable due to the wide variability in the targeted population or sub-population of each study. If no sample size calculations were reported, “unclear” was given as an answer to this question.

**^§^** The following diagnostic techniques were decided to provide a reliable measurement of DENV sero-prevalence: Enzyme-Linked Immuno-Sorbent Assay (ELISA), ImmunoFluorescence Assay (IFA), Reverse-Transcriptase Polymerase Chain Reaction (RT-PCR) and Viral Neutralization Tests (VNT).

**^¶^** Important confounding factors were considered to be properly addressed if the study findings were confirmed by PCR or an investigation for cross-reactivity against other arboviral infection markers was conducted.

**References**

1. Sharp TM, Moreira R, Soares MJ, Miguel da Costa L, Mann J, DeLorey M, et al. Underrecognition of Dengue during 2013 Epidemic in Luanda, Angola. Emerg Infect Dis [Internet]. 2015 Aug 23; 21(8):1311–6.
2. Lim JK, Seydou Y, Carabali M, Barro A, Dahourou DL, Lee KS, et al. Clinical and epidemiologic characteristics associated with dengue during and outside the 2016 outbreak identified in health facility-based surveillance in Ouagadougou, Burkina Faso. PLoS Negl Trop Dis. 2019 Dec 6;13(12):e0007882. doi: 10.1371/journal.pntd.0007882
3. Ridde V, Agier I, Bonnet E, Carabali M, Dabiré KR, Fournet F, et al. Presence of three dengue serotypes in Ouagadougou (Burkina Faso): Research and public health implications. Infect Dis Poverty. 2016 Dec;5(1):1-3.
4. Sawadogo S, Baguiya A, Yougbare F, Bicaba BW, Nebie K, Millogo T, et al. Seroprevalence and factors associated with IgG anti-DENV positivity in blood donors in Burkina Faso during the 2016 dengue outbreak and implications for blood supply. Transfus Med. 2020 Feb 1;30(1):37–45.
5. Tarnagda Z, Cissé A, Bicaba BW, Diagbouga S, Sagna T, Ilboudo AK, et al. Dengue Fever in Burkina Faso, 2016. Emerg Infect Dis. 2018 Jan 1;24(1):170–2.
6. Mouiche MMM, Ntumvi NF, Maptue VT, Tamoufe U, Albert B, Ngum Ndze V, et al. Evidence of Low-Level Dengue Virus Circulation in the South Region of Cameroon in 2018. Vol. 20, Vector-Borne and Zoonotic Diseases. Mary Ann Liebert Inc.; 2020 Apr 1;20(4):314-7.
7. Nemg Simo FB, Sado Yousseu FB, Evouna Mbarga A, Bigna JJ, Melong A, Ntoude A, et al. Investigation of an Outbreak of Dengue Virus Serotype 1 in a Rural Area of Kribi, South Cameroon: A Cross-Sectional Study. Intervirology. 2019 Jun 1;61(6):265–71. doi: 10.1159/000499465
8. Tchuandom SB, Tchadji JC, Tchouangueu TF, Biloa MZ, Atabonkeng EP, Fumba MIM, et al. A cross-sectional study of acute dengue infection in paediatric clinics in Cameroon. BMC Public Health. 2019 Dec;19(1):1-7.
9. Yousseu FBS, Nemg FBS, Ngouanet SA, Mekanda FMO, Demanou M. Detection and serotyping of dengue viruses in febrile patients consulting at the New-Bell District Hospital in Douala, Cameroon. PLoS One. 2018 Oct 3;13(10):e0204143. doi: 10.1371/journal.pone.0204143
10. Dellagi K, Salez N, Maquart M, Larrieu S, Yssouf A, Silaï R, et al. Serological Evidence of Contrasted Exposure to Arboviral Infections between Islands of the Union of Comoros (Indian Ocean). PLoS Negl Trop Dis. 2016 Dec 15;10(12):e0004840. doi: 10.1371/journal.pntd.0004840.
11. Moyen N, Thiberville SD, Pastorino B, Nougairede A, Thirion L, Mombouli JV, et al. First reported chikungunya fever outbreak in the republic of Congo, 2011. PLoS One. 2014 Dec 26;9(12):e115938. doi: 10.1371/journal.pone.0115938.
12. L’Azou M, Succo T, Kamagaté M, Ouattara A, Gilbernair E, Adjogoua E, et al. Dengue: etiology of acute febrile illness in Abidjan, Côte d’Ivoire, in 2011-2012. Trans R Soc Trop Med Hyg. 2015 Nov;109(11):717–22.
13. Proesmans S, Katshongo F, Milambu J, Fungula B, Mavoko HM, Ahuka-Mundeke S, da Luz RI, Van Esbroeck M, Ariën KK, Cnops L, De Smet B. Dengue and chikungunya among outpatients with acute undifferentiated fever in Kinshasa, Democratic Republic of Congo: A cross-sectional study. PLoS neglected tropical diseases. 2019 Sep 5;13(9):e0007047. doi: 10.1371/journal.pntd.0007047.
14. Willcox AC, Collins MH, Jadi R, Keeler C, Parr JB, Mumba D, et al. Seroepidemiology of dengue, zika, and yellow fever viruses among children in the democratic republic of the Congo. Am J Trop Med Hyg. 2018;99(3):756–63.
15. Degife LH, Worku Y, Belay D, Bekele A, Hailemariam Z. Factors associated with dengue fever outbreak in Dire Dawa administration city, October, 2015, Ethiopia - Case control study. BMC Public Health. 2019 Dec;19(1):1-7.
16. Ferede G, Tiruneh M, Abate E, Wondimeneh Y, Damtie D, Gadisa E, et al. A serologic study of dengue in northwest Ethiopia: Suggesting preventive and control measures. PLoS Negl Trop Dis. 2018 May 31;12(5):e0006430. doi: 10.1371/journal.pntd.0006430.
17. Geleta EN. Serological evidence of dengue fever and associated factors in health facilities. bioRxiv. Cold Spring Harbor Laboratory; 2018 Dec. Available at: <https://www.biorxiv.org/content/10.1101/502617v1.> doi: 10.1101/502617.
18. Tsegaye MM, Beyene B, Ayele W, Abebe A, Tareke I, Sall A, Yactayo S, Shibeshi ME, Staples E, Belay D, Lilay A. Sero-prevalence of yellow fever and related Flavi viruses in Ethiopia: a public health perspective. BMC public health. 2018 Dec 1;18(1):1-10. doi: 10.1186/s12889-018-5726-9
19. Abe H, Ushijima Y, Loembe MM, Bikangui R, Nguema-Ondo G, Mpingabo PI, et al. Re-emergence of dengue virus serotype 3 infections in Gabon in 2016–2017, and evidence for the risk of repeated dengue virus infections. Int J Infect Dis. 2020 Feb 1;91:129–36.
20. Nkoghe D, Kassa RFK, Bisvigou U, Caron M, Grard G, Leroy EM. No clinical or biological difference between Chikungunya and Dengue Fever during the 2010 Gabonese outbreak. Infect Dis Rep. 2012 Jan;4(1):11-3.
21. Amoako N, Duodu S, Dennis FE, Bonney JHK, Asante KP, Ameh J, et al. Detection of dengue virus among children with suspected malaria, Accra, Ghana. Emerg Infect Dis. 2018 Aug 1;24(8):1561–4.
22. Bonney JHK, Hayashi T, Dadzie S, Agbosu E, Pratt D, Nyarko S, et al. Molecular detection of dengue virus in patients suspected of Ebola virus disease in Ghana. PLoS One. 2018 Dec 19;13(12):e0208907. doi: 10.1371/journal.pone.0208907.
23. Manu SK, Bonney JHK, Pratt D, Abdulai FN, Agbosu EE, Frimpong PO, et al. Arbovirus circulation among febrile patients at the greater Accra Regional Hospital, Ghana. BMC Res Notes. 2019 Dec;12(1):1-5.
24. Stoler J, Delimini RK, Kofi Bonney JH, Oduro AR, Owusu-Agyei S, Fobil JN, et al. Evidence of recent dengue exposure among malaria parasite-positive children in three urban centers in Ghana. Am J Trop Med Hyg. 2015 Mar 1;92(3):497–500.
25. Blaylock JM, Maranich A, Bauer K, Nyakoe N, Waitumbi J, Martinez LJ, et al. The seroprevalence and seroincidence of dengue virus infection in western Kenya. Travel Med Infect Dis. 2011 Sep;9(5):246–8.
26. Chepkorir E, Tchouassi DP, Konongoi SL, Lutomiah J, Tigoi C, Irura Z, et al. Serological evidence of Flavivirus circulation in human populations in Northern Kenya: An assessment of disease risk 2016-2017. Virol J. 2019 Dec;16(1):1-10.
27. Ellis EM, Neatherlin JC, Delorey M, Ochieng M, Mohamed AH, Mogeni DO, et al. A Household Serosurvey to Estimate the Magnitude of a Dengue Outbreak in Mombasa, Kenya, 2013. PLoS Negl Trop Dis. 2015 Apr 29;9(4):e0003733. doi: 10.1371/journal.pntd.0003733.
28. Grossi-Soyster EN, Cook EAJ, de Glanville WA, Thomas LF, Krystosik AR, Lee J, et al. Serological and spatial analysis of alphavirus and flavivirus prevalence and risk factors in a rural community in western Kenya. PLoS Negl Trop Dis. 2017 Oct 17;11(10):e0005998. doi: 10.1371/journal.pntd.0005998.
29. Inziani M, Adungo F, Awando J, Kihoro R, Inoue S, Morita K, et al. Seroprevalence of yellow fever, dengue, West Nile and chikungunya viruses in children in Teso South Sub-County, Western Kenya. Int J Infect Dis. 2020 Feb 1;91:104–10.
30. Konongoi SL, Nyunja A, Ofula V, Owaka S, Koka H, Koskei E, et al. Human and entomologic investigations of chikungunya outbreak in Mandera, Northeastern Kenya, 2016. PLoS One. 2018 Oct 11;13(10):e0205058. doi: 10.1371/journal.pone.0205058.
31. Konongoi L, Ofula V, Nyunja A, Owaka S, Koka H, Makio A, et al. Detection of dengue virus serotypes 1, 2 and 3 in selected regions of Kenya: 2011-2014. Virol J. 2016 Nov 4;13(1):1–11.
32. Masika MM, Korhonen EM, Smura T, Uusitalo R, Vapalahti K, Mwaengo D, et al. Detection of dengue virus type 2 of Indian origin in acute febrile patients in rural Kenya. PLoS Negl Trop Dis. 2020 Mar 3;14(3):e0008099. doi: 10.1371/journal.pntd.0008099.
33. Ngoi CN, Price MA, Fields B, Bonventure J, Ochieng C, Mwashigadi G, et al. Dengue and Chikungunya Virus Infections among Young Febrile Adults Evaluated for Acute HIV-1 Infection in Coastal Kenya. PLOS One. 2016 Dec 12;11(12):e0167508. doi: 10.1371/journal.pone.0167508.
34. Obonyo M, Fidhow A, Ofula V. Investigation of laboratory confirmed dengue outbreak in north-eastern Kenya, 2011. PLoS ONE. 2018 Jun 7;13(6):e0198556. doi: 10.1371/journal.pone.0198556.
35. Vu DM, Mutai N, Heath CJ, Vulule JM, Mutuku FM, Ndenga BA, et al. Unrecognized dengue virus infections in children, Western Kenya, 2014-2015. Emerg Infect Dis. 2017 Nov;23(11):1915-7. doi: 10.3201/eid2311.170807.
36. Vu DM, Banda T, Teng CY, Heimbaugh C, Muchiri EM, Mungai PL, et al. Dengue and west Nile virus transmission in children and adults in coastal Kenya. Am J Trop Med Hyg. 2017 Jan 1;96(1):141–3.
37. Schwarz NG, Girmann M, Randriamampionona N, Bialonski A, Maus D, Krefis AC, et al. Seroprevalence of antibodies against Chikungunya, Dengue, and Rift Valley fever viruses after febrile illness outbreak, Madagascar. Emerg Infect Dis. 2012 Nov ;18(11):1780–6.
38. Massangaie M, Pinto G, Padama F, Chambe G, Da Silva M, Mate I, et al. Clinical and epidemiological characterization of the first recognized outbreak of dengue virus-type 2 in Mozambique, 2014. Am J Trop Med Hyg. 2016 Feb 1;94(2):413–6.
39. Mugabe VA, Ali S, Chelene I, Monteiro VO, Guiliche O, Muianga AF, et al. Evidence for chikungunya and dengue transmission in Quelimane, Mozambique: Results from an investigation of a potential outbreak of chikungunya virus. PLoS One. 2018 Feb 7;13(2):e0192110. doi: 10.1371/journal.pone.0192110.
40. Muianga A, Pinto G, Massangaie M, Ali S, Oludele J, Tivane A, et al. Antibodies against chikungunya in northern Mozambique during dengue outbreak, 2014. Vector-Borne Zoonotic Dis. 2018 Aug 1;18(8):445–9.
41. Noden BH, Musuuo M, Aku-Akai L, Van der Colf B, Chipare I, Wilkinson R. Risk assessment of flavivirus transmission in Namibia. Acta Trop. 2014;137:123–9.
42. Ayolabi CI, Olusola BA, Ibemgbo SA, Okonkwo GO. Detection of Dengue viruses among febrile patients in Lagos, Nigeria and phylogenetics of circulating Dengue serotypes in Africa. Infection, Genetics and Evolution. 2019 Nov 1;75:103947. doi: 10.1016/j.meegid.2019.103947.
43. Idoko MO, Ado SA, Umoh VJ. Prevalence of Dengue Virus and Malaria in Patients with Febrile Complaints in Kaduna Metropolis, Nigeria. Br Microbiol Res J Int. 2015 Apr 10;343–7.
44. Kolawole OM, Seriki AA, Irekeola AA, Bello KE, Adeyemi OO. Dengue virus and malaria concurrent infection among febrile subjects within Ilorin metropolis, Nigeria. J Med Virol. 2017 Aug 1;89(8):1347–53.
45. Moses AE, Atting IA, Inyang OS. Evidence of overlapping infections of dengue, malaria and typhoid in febrile patients attending a tertiary health facility in Uyo, South-South Nigeria. Journal of Advances in Medicine and Medical Research. 2016 Aug 3;17(3):1-9.
46. Mustapha JO, Emeribe AU, Nasir IA. Survey of malaria and anti-dengue virus IgG among febrile HIV-infected patients attending a tertiary hospital in Abuja, Nigeria. HIV/AIDS - Res Palliat Care. 2017 Jun 30;9:145–51.
47. Nasir IA, Agbede OO, Dangana A, Baba M, Haruna AS. Dengue virus non-structural Protein-1 expression and associated risk factors among febrile Patients attending University of Abuja Teaching Hospital, Nigeria. Virus Res. 2017 Feb 15;230:7–12.
48. Omatola CA, Onoja AB, Moses E, Mahmud M, Mofolorunsho CK. Dengue in parts of the Guinea Savannah region of Nigeria and the risk of increased transmission. Int Health. 2020 Jun 20. doi: 10.1093/inthealth/ihaa033.
49. Onoja AB, Adeniji JA, Olaleye OD. High rate of unrecognized dengue virus infection in parts of the rainforest region of Nigeria. Acta Trop. 2016 Aug 1;160:39–43.
50. Onyedibe K, Dawurung J, Iroezindu M, Shehu N, Okolo M, Shobowale E, et al. A cross sectional study of dengue virus infection in febrile patients presumptively diagnosed of malaria in Maiduguri and Jos Plateau, Nigeria. Malawi Med J. 2018 Dec 1;30(4):276–82.
51. Otu AA, Udoh UA, Ita OI, Hicks JP, Egbe WO, Walley J. A cross-sectional survey on the seroprevalence of dengue fever in febrile patients attending health facilities in Cross River State, Nigeria. PLoS One. 2019 Apr 22;14(4):e0215143. doi: 10.1371/journal.pone.0215143.
52. Oyero OG, Ayukekbong JA. High dengue NS1 antigenemia in febrile patients in Ibadan, Nigeria. Virus Res. 2014 Oct 13;191(1):59–61.
53. Sule WF, Fadamitan TO, Lawal OA, Adebimpe WO, Opaleye OO, Oluwayelu DO. Probable primary and secondary dengue viral infections and associated host factors among university undergraduates in Osun State, Nigeria. Alexandria J Med. 2019 Jan 2;55(1):25–30.
54. Dieng I, Hedible BG, Diagne MM, El Wahed AA, Diagne CT, Fall C, et al. Mobile Laboratory Reveals the Circulation of Dengue Virus Serotype I of Asian Origin in Medina Gounass (Guediawaye), Senegal. Diagnostics. 2020 Jun;10(6):408. doi: 10.3390/diagnostics10060408.
55. Sokhna C, Mediannikov O, Fenollar F, Bassene H, Diatta G, Tall A, et al. Point-of-Care Laboratory of Pathogen Diagnosis in Rural Senegal. PLoS Negl Trop Dis. 2013 Jan 17;7(1):e1999. doi: 10.1371/journal.pntd.0001999.
56. Sow A, Loucoubar C, Diallo D, Faye O, Ndiaye Y, Senghor CS, et al. Concurrent malaria and arbovirus infections in Kedougou, southeastern Senegal. Malar J. 2016 Dec;15(1):1-7.
57. Boisen ML, Schieffelin JS, Goba A, Oottamasathien D, Jones AB, Shaffer JG, et al. Multiple circulating infections can mimic the early stages of viral hemorrhagic fevers and possible human exposure to filoviruses in sierra leone prior to the 2014 outbreak. Viral Immunol. 2015 Feb 1;28(1):19–31.
58. Dariano DF, Taitt CR, Jacobsen KH, Bangura U, Bockarie AS, Bockarie MJ, et al. Surveillance of vector-borne infections (chikungunya, dengue, and malaria) in Bo, Sierra Leone, 2012-2013. Am J Trop Med Hyg. 2017;97(4):1151–4.
59. de Araújo Lobo JM, Mores CN, Bausch DG, Christofferson RC. Short Report: Serological Evidence of Under-Reported Dengue Circulation in Sierra Leone. PLoS Negl Trop Dis. 2016 Apr 26;10(4):e0004613. doi: 10.1371/journal.pntd.0004613.
60. Abdalla TM, Karsany MS, Ali AA. Correlation of measles and dengue infection in Kassala, Eastern Sudan. J Med Virol. 2015 Jan 1;87(1):76–8.
61. Abdallah TM, Ali AAA, Karsany MS, Adam I. Epidemiology of dengue infections in Kassala, Eastern Sudan. J Med Virol. 2012 Mar ;84(3):500–3.
62. Adam A, Schüttoff T, Reiche S, Jassoy C. High seroprevalence of dengue virus indicates that dengue virus infections are frequent in central and eastern Sudan. Trop Med Int Heal. 2018 Sep 1;23(9):960–7.
63. Ahmed A, Elduma A, Magboul B, Higazi T, Ali Y. The First Outbreak of Dengue Fever in Greater Darfur, Western Sudan. Trop Med Infect Dis. 2019 Mar;4(1):43. doi: 10.3390/tropicalmed4010043.
64. Eldigail MH, Adam GK, Babiker RA, Khalid F, Adam IA, Omer OH, et al. Prevalence of dengue fever virus antibodies and associated risk factors among residents of El-Gadarif state, Sudan. BMC Public Health. 2018 Dec;18(1):1-8.
65. Eldigail MH, Abubaker HA, Khalid FA, Abdallah TM, Adam IA, Adam GK, et al. Recent transmission of dengue virus and associated risk Facors among residents of Kassala state, eastern Sudan. BMC Public Health. 2020 Dec;20:1-9.
66. Elduma AH, Osman WM. Dengue and hepatitis E virus infection in pregnant women in Eastern Sudan, A challenge for diagnosis in an endemic area. Pan Afr Med J. 2014 Dec 18;19:391. doi: 10.11604/pamj.2014.19.391.5439
67. Hamid Z, Hamid T, Alsedig K, Abdallah T, Elaagip A, Ahmed A, et al. Molecular investigation of dengue virus serotype 2 circulation in Kassala state, Sudan. Jpn J Infect Dis. 2019;72(1):58–61.
68. Himatt S, Osman KE, Okoued SI, Seidahmed OE, Beatty ME, Soghaier MA, et al. Sero-prevalence of dengue infections in the Kassala state in the eastern part of the Sudan in 2011. Journal of Infection and Public Health. 2015 Sep 1;8(5):487-92.
69. Soghaier MA, Mahmood SF, Pasha O, Azam SI, Karsani MM, Elmangory MM, et al. Factors associated with dengue fever IgG sero-prevalence in South Kordofan State, Sudan, in 2012: Reporting prevalence ratios. J Infect Public Health. 2014 Feb;7(1):54–61.
70. Soghaier MA, Himatt S, Osman KE, Okoued SI, Seidahmed OE, Beatty ME, et al. Cross-sectional community-based study of the socio-demographic factors associated with the prevalence of dengue in the eastern part of Sudan in 2011 Infectious Disease epidemiology. BMC Public Health. 2015 Dec;15(1):1-6. doi: 10.1186/s12889-015-1913-0
71. Boillat-Blanco N, Mbarack Z, Samaka J, Mlaganile T, Mamin A, Genton B, et al. Prognostic value of quickSOFA as a predictor of 28-day mortality among febrile adult patients presenting to emergency departments in Dar es Salaam, Tanzania. PLoS One. 2018 Jun 14;13(6):e0197982. doi: 10.1371/journal.pone.0197982.
72. Budod RM, Horumpende PG, Mkumbaye SI, Mmbaga BT, Mwakapuja RS, Chilongola JO. Serological evidence of exposure to Rift Valley, Dengue and Chikungunya Viruses among agropastoral communities in Manyara and Morogoro regions in Tanzania: A community Survey. bioRxiv. 2020 Jan 16;2020.01.16.908830. doi: 10.1101/2020.01.16.908830.
73. Chipwaza B, Mugasa JP, Selemani M, Amuri M, Mosha F, Ngatunga SD, et al. Dengue and Chikungunya Fever among Viral Diseases in Outpatient Febrile Children in Kilosa District Hospital, Tanzania. PLoS Negl Trop Dis. 2014 Nov 20;8(11):e3335. doi: 10.1371/journal.pntd.0003335.
74. Faustine NL, Sabuni EJ, Ndaro AJ, Paul E, Chilongola JO. Chikungunya, Dengue and West Nile Virus Infections in Northern Tanzania. J Adv Med Med Res. 2017 Oct 26;24(4):1-7.
75. Hercik C, Cosmas L, Mogeni OD, Wamola N, Kohi W, Houpt E, et al. A combined syndromic approach to examine viral, bacterial, and parasitic agents among febrile patients: A pilot study in Kilombero, Tanzania. Am J Trop Med Hyg. 2018;98(2):625–32.
76. Kajeguka DC, Kaaya RD, Mwakalinga S, Ndossi R, Ndaro A, Chilongola JO, et al. Prevalence of dengue and chikungunya virus infections in north-eastern Tanzania: A cross sectional study among participants presenting with malaria-like symptoms. BMC Infect Dis. 2016 Dec;16(1):1-9.
77. Kajeguka DC, Kaaya RD, Desrochers R, Iranpour M, Kavishe RA, Mwakalinga S, et al. Mapping clusters of chikungunya and dengue transmission in northern Tanzania using disease exposure and vector data. Tanzania Journal of Health Research. 2017;19(4). doi: 10.4314/thrb.v19i4..
78. Mwanyika GO, Mboera L, Rugarabamu S, Lutwama J, Sindato C, Paweska J, et al. Co-circulation of Dengue Virus Serotypes 1 and 3 during the 2019 epidemic in Dar es Salaam, Tanzania. bioRxiv. 2019 Sep 9;5:763003. doi: 10.1101/763003.
79. Saro A, Kajeguka D, Azizi K, Mwakalinga S, Mosha F, Alifrangis M, et al. Chikungunya and dengue virus infection among febrile children in North-Eastern Tanzania: prospective study. Researchsquare. 2020. doi: 10.21203/rs.3.rs-22979/v1.
80. Vairo F, Nicastri E, Yussuf SM, Cannas A, Meschi S, Mahmoud MAA, et al. IgG against dengue virus in healthy blood donors, Zanzibar, Tanzania. Emerg Infect Dis. 2014 Mar;20(3):465–8.
81. Vairo F, Mboera LEG, de Nardo P, Oriyo NM, Meschi S, Rumisha SF, et al. Clinical, virologic, and epidemiologic characteristics of dengue outbreak, Dar es Salaam, Tanzania, 2014. Emerg Infect Dis. 2016 May 1;22(5):895–9.
82. Kayiwa JT, Nankya AM, Ataliba IJ, Mossel EC, Crabtree MB, Lutwama JJ. Confirmation of Zika virus infection through hospital-based sentinel surveillance of acute febrile illness in Uganda, 2014–2017. The Journal of general virology. 2018 Sep;99(9):1248-52.
83. Chisenga CC, Bosomprah S, Musukuma K, Mubanga C, Chilyabanyama ON, Velu RM, et al. Sero-prevalence of arthropod-borne viral infections among Lukanga swamp residents in Zambia. PLoS One. 2020 Jul 1;15(7):e0235322. doi: 10.1371/journal.pone.0235322
84. Mazaba-Liwewe ML, Babaniyi O, Monza M, Mweene-Ndumba I, Mulenga D, Masaninga F, et al. Dengue fever and factors associated with it in Western provinces of Zambia. International Public Health Journal. 2016;8(1):65.
